# Graft-versus-host-disease after kidney transplantation: A case report and literature review

**DOI:** 10.1097/MD.0000000000007333

**Published:** 2017-06-30

**Authors:** Yanhua Guo, Shouyang Ding, Hui Guo, Shenqiu Li, Xia Lu, Zhishui Chen, Zhonghua Klaus Chen, Changsheng Ming, Nianqiao Gong

**Affiliations:** a Institute of Organ Transplantation, Tongji Hospital, Tongji Medical College, Huazhong University of Science and Technology, Wuhan; b Department of Transplantation, Central Hospital of Yiyang, Yiyang; c Department of Dermatology, Tongji Hospital, Tongji Medical College, Huazhong University of Science and Technology, Wuhan, China.

**Keywords:** chimerism, graft-versus-host-disease (GVHD), immunosuppression, kidney transplantation, pathology

## Abstract

**Introduction:**

Acute graft-versus-host-disease (GVHD) in kidney recipients is extremely rare. Knowledge about its clinical manifestations, diagnosis, treatment, and prognosis is limited and needs to be increased.

**Clinical Findings:**

One male kidney transplant recipient developed diarrhea and suffered kidney function damage. Primarily diagnosed with acute rejection, he was given methylprednisolone (MP) bolus treatment. Meanwhile, intravenous immunoglobulin (IVIG) and decreased immunosuppressive agents were applied for the corresponding infection. During the treatment, skin rashes occurred over his whole body. Biopsies were then taken. The pathology of the kidney graft showed no rejection, while the skin pathology revealed typical GVHD. Furthermore, fluorescence in situ hybridization proved the presence of donor-derived cells in the skin lesions, and infiltrating cytotoxic T cells and NK cells were identified in the rash.

**Outcome:**

Based on the clinical presentations, pathological findings, and chimerism detection, GVHD after kidney transplantation was confirmed as the final diagnosis. The recipient responded well to treatment. His kidney function recovered, and the skin lesions were completely resolved. He has been followed for 1 year without any further episodes.

**Conclusion:**

GVHD after kidney transplantation has its own characteristics. In the presence of a highly immunocompromised state, diarrhea and rashes, a diagnosis of GVHD needs to be considered. Kidney function impairment may be involved. Pathological changes and detection of chimerism and immunocyte infiltration are required for diagnosis. MP bolus, IVIG, and decreased immunosuppression could be beneficial to the clinical outcome. Kidney recipients have a prognosis superior to recipients of organs bearing large numbers of lymphocytes.

## 1 Introduction

Acute graft-versus-host-disease (GVHD) is a rare but fatal complication following transplantation.^[1]^ After liver transplantation, acute GVHD occurs with an incidence of 0.1% to 1%^[2]^ and the mortality rate exceeds 75%.^[3]^ The onset of GVHD needs functioning donor-derived T cells that cause a local inflammatory reaction.^[4]^ Generally, the transplant requires a large volume or enrichment of lymphocytes. For example, a liver has 10^9^ donor-derived lymphocytes to transfer to the recipient.^[5]^ GVHD also occurs after other kinds of solid organ transplantation such as lung, intestine, and pancreas-kidney.^[6–8]^ The occurrence of GVHD after kidney transplantation is extremely rare. To date, only 5 cases have been reported.^[9–13]^ With so few cases, knowledge about clinical manifestation, diagnosis, treatment, and prognosis of GVHD after kidney transplantation is limited and needs to be increased.

## 2 Case presentation

A 57-year-old man underwent kidney transplantation due to uremia. The kidney was procured from a donor who was brain dead due to a cerebrovascular accident. The donor's HLA typing was A:2,-; B:13,15; DR:7,15 while the recipient's was A:2,11; B:13,60; DR:7,8. The recipient's panel reactive antibody (PRA) level before the transplant was negative. The cross-match was negative. The donation and transplantation were approved by the committee of ethics at Central Hospital of Yiyang.

The recipient received induction therapy with rabbit-antithymocyte globulin 100 mg/d for 6 days and a triple immunosuppressive regimen including tacrolimus (Tac) + Mycophenolate mofetil (MMF) + prednisone (Pred). On POD20, his serum creatinine (Scr) level was 113 μmol/L. On POD28, the Scr increased to 203 μmol/L while the Tac trough level was 10.6 ng/L. Given concerns about tacrolimus-related renal toxicity and rejection due to the decreased Tac level, the recipient's immunosuppressive regimen was converted to low-dose Tac + rapamycin + MMF + Pred in a local follow-up clinic. One week later, the Scr was 126 μmol/L with the Tac trough level at 10.6 ng/L and rapamycin 1 mg Qd. On POD37, the recipient developed a cough, expectoration, decreased appetite, diarrhea, and cachexia, with urine output decreased to 600 mL. A sputum smear indicated the presence of fungi in the sputum. Afterward, the recipient was treated with voriconazole 200 mg/d orally.

On POD41, the recipient was admitted to our institute. His urine output was 100 mL and his Scr was 316 μmol/L. His liver function was normal. His blood work showed WBC 5.21×10^9^/L (N: 92.7%), HB 80.0 g/L, and platelet count 176.0 × 10^9^/L. The Tac trough level was 5.4 ng/L and MPA AUC was 91.73 mg × h/L. Based on an increased Scr, decreased urine output, and a relatively low Tac trough level, the patient's primary diagnosis was acute rejection. Following an immediate renal biopsy, MP bolus treatment was given: 500 mg/d for 2 days, 300 mg on the third day, and 20 mg/d for maintenance. Meanwhile, to treat the diarrhea and cachexia, the patient was given fluid treatment and nutritional support. He responded well, with his urine output increasing to 1000 mL within 1 day. The output subsequently increased to more than 2000 mL per day, and the Scr gradually decreased to a normal range within 10 days.

On the second day after admission (POD42), a lung CT showed interstitial pneumonia. In addition to MP bolus treatment, Tac was thereafter maintained at a trough level of 5 to 6 ng/mL, and MMF and rapamycin were withdrawn immediately. IVIG at 10 g/d was given for 10 doses. Moreover, a broad-spectrum antibiotic was administered for 29 days. To treat the possible pathogens that may have induced the interstitial pneumonia, gancyclovir was used for 14 days (without positive virus pathogen findings) and trimethoprim-sulfamethoxazole was given on POD45 for 90 days against a possible pneumocystis carinii infection that may have been related to rapamycin. Additionally, micafungin sodium was given for 23 days although neither aspergillus nor candida was identified by fungi culture or CT scan.

**Figure 1:**
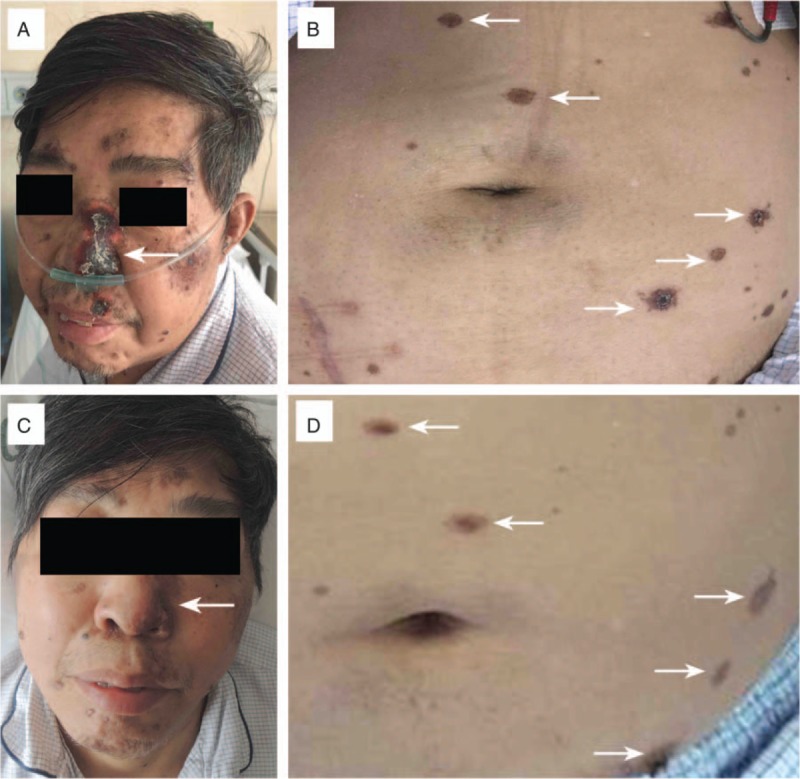
Development of skin rashes after kidney transplantation. A and B, Three weeks after occurrence, skin rashes (arrow) were distributed on the face and abdomen. C and D, Six weeks after occurrence, skin rashes (arrow) were resolving.

Also on POD42, rashes developed sporadically on the recipient's face and back. A rash biopsy was taken. Thereafter, more rashes were distributed over his whole body, mainly on the face and abdomen (Fig. 1A and B). Seven days after occurrence, the rashes began to scab and were completely resolved by POD90.

**Figure 2:**
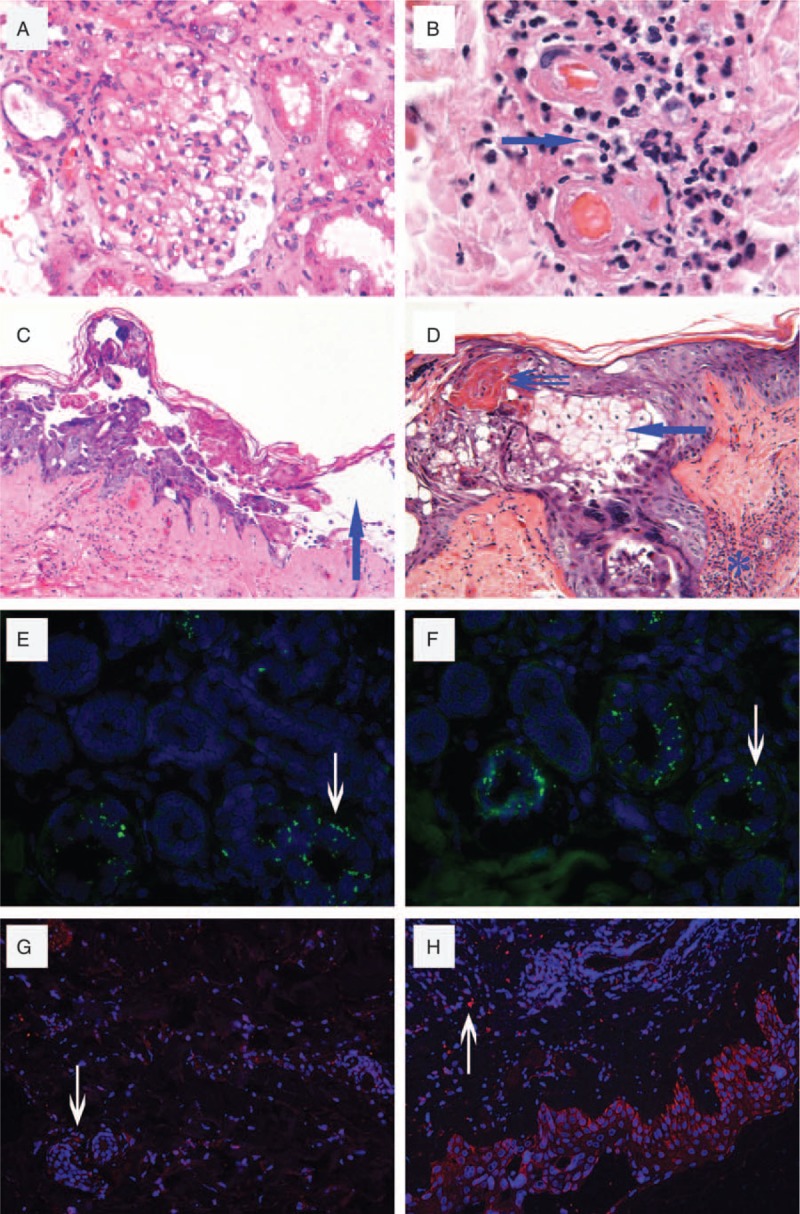
Detection on the kidney graft and skin rash. A, Renal allograft biopsy showed normal structure and no signs of allograft rejection. H&E, ×200. B, Grade III acute GVHD: peripheral lymphocytes infiltrating around the branches of the arteriole (arteriole vasculitis in the dermis). H&E, ×200. C, Grade III acute GVHD: necrosis of basal cells and acanthocytes in the basal cells resulting in cleft formation; more superficial layers resulting in separation and demo-epidermal junction (arrowhead). H&E, ×100. D, Grade III acute GVHD: the focal spongiosis and dyskeratosis (arrow), eosinophilic necrosis of the epidermal cells (double arrows), and moderated mononuclear infiltration of the papillary dermis (aster). H&E, ×200. E, B15 locus in the nucleus was stained by a specific DNA probe with green fluorescence (arrow). FISH ×400. F, DR15 locus in the nucleus was stained (arrow). FISH ×400. G, CD8 + cytotoxic T cells were shown with red fluorescence in (arrow). immunofluorescence, ×200. H, CD56 + NK cells were shown (arrow). Immunofluorescence, ×200. FISH = fluorescence in situ hybridization, GVHD = acute graft-versus-host-disease.

On POD47, the pathological results were reported. Strikingly, the renal pathology gave a normal finding without any sign of rejection (Fig. 2A). The rash pathology showed typical grade III acute GVHD based on the histology criteria:^[8]^ peripheral lymphocytes infiltrating around the branches of the arteriole (arteriole vasculitis in the dermis); necrosis of the basal cells and cleft formation; acanthocytes in the basal cells; more superficial layers resulting in separation and demo-epidermal junction; focal spongiosis, dyskeratosis and eosinophilic necrosis of the epidermal cells; and moderate mononuclear infiltration of the papillary dermis (Fig. 2B–D). The rash tissue was detected by fluorescence in situ hybridization (FISH), and the presence of donor-derived HLA loci B15^+^ and DR1^+^ cells was determined (Fig. 2E and F). Moreover, infiltrating cytotoxic CD8^+^T cells and CD56^+^ NK cells were identified in the skin rashes by immunofluorescence (Fig. 2G and H).

On POD60, the recipient's clinical symptoms recovered; the sputum, blood, and virus assays all reported negative, and he was returned to a triple immunosuppressive regimen (Tac + MMF + Pred) with regular Tac trough level monitoring (8–10 ng/dL). On POD90, his Scr was 132 μmol/L, and the skin lesions were completely resolved (Fig. 1C and D). He was followed for 1 year without any further episodes.

## 3 Discussion

Before the onset of GVHD, the recipient had received ATG induction + classical triple immunosuppression and even a short-term quadruple regime. Although his Tac trough level showed a temporary decrease, the patient generally experienced a highly immunocompromised state that may have been responsible for the following pneumonia, suggesting a susceptibility to GVHD.^[14]^

The diagnosis of GVHD of the recipient was delayed, as happened in each of the 5 reported cases, although the patients were showing typical manifestations (diarrhea and rashes). Limited experience and lack of awareness were the main reasons. Another reason was that the recipient's primary diagnosis was thought to be acute rejection because of the temporarily low Tac trough level and kidney damage (urine output decrease and Scr increase). Retrospectively, 2 of the 5 reported cases had already experienced kidney damage, which seems to be a marked characteristic of GVHD after kidney transplantation. Interestingly, a kidney biopsy proved no rejection, and a skin biopsy exhibited typical pathological features of GVHD. Based on the clinical presentations and pathological findings, the diagnosis of GVHD was formed.

Notably, the differential diagnosis of toxic epidermal necrolysis (TEN) needs to be excluded. TEN could happen under exposure to medicines such as anti-infection drugs or immunosuppressive agents, with a pathological presentation of separation at the dermo-epidermal junction. These TEN features also existed in the current case. Therefore, we further investigated 3 supportive evidences for GVHD. First, the pathology identified the manifestation of arteriole vasculitis in the dermis. This only occurs under immune response such as acute rejection but not TEN. Second, our case is the first kidney recipient diagnosed as GVHD based on the findings of chimerism of donor-derived cells in the recipient's tissue (skin rash). All of the prior GVHD diagnoses on kidney recipients were based only on the integration of clinical findings and biopsy pathology,^[9–13]^ although FISH or short tandem repeat (STR) has been used for chimerism detection in liver and pancreas transplantation.^[15,16]^ Third, we identified the infiltration of cytotoxic T cells and NK cells in the lesions, which points to an ongoing cellular immunity but not TEN. Combined with the clinical presentations, pathological findings, and chimerism detection, the diagnosis of GVHD in this case was finally confirmed.

The recipient received treatments including MP bolus, IVIG, decreased immunosuppression, and anti-infection medication. The therapy was aimed to treat acute rejection and infection. Fortunately, he obtained a quick response because this strategy is also effective on GVHD. The reasons for reversion to GVHD are deduced as follows: MP bolus treatment destroyed the activated donor-derived lymphocytes; decreased immunosuppression was beneficial to delete donor-derived lymphocytes by the host's native immune system; IVIG may have modulated the immune reaction while also having an effect on the infection. The recovery of kidney function should have been due to the fluid treatment and the adjustment of gastrointestinal disorder-related immunosuppressants that corrected the diarrhea-induced hypovolemia, which only resulted in transient acute kidney injury without pathological changes.

The prognosis of GVHD after kidney transplantation is relatively superior to that after other solid transplantation. To date, only 2 of 6 kidney recipients have died, compared with 75% of liver recipients.^[3]^ The reason for this may be that a kidney graft bears much fewer passenger lymphocytes than the liver or other organs. The outcome also could be partly related to timely clinical diagnosis and treatments.

In conclusion, the current case shows specific characteristics of GVHD after kidney transplantation: with the presence in the recipient of a highly immunocompromised state, diarrhea and rashes, the diagnosis of GVHD needs to be considered; kidney function impairment may be involved; besides clinical manifestations and pathological changes, chimerism and immunocyte infiltrating should be detected; MP bolus, IVIG, and decreased immunosuppression could be beneficial to the clinical outcome; kidney recipients have a prognosis superior to recipients of organs bearing large numbers of lymphocytes.

## References

[1] Sharma A Armstrong AE Posner MP Graft-versus-host disease after solid organ transplantation: a single center experience and review of literature. Ann Transplant 2012;17:133–9. 10.12659/aot.883704 23274334

[2] Jeanmonod P Hubbuch M Grunhage F Graft-versus-host disease or toxic epidermal necrolysis: diagnostic dilemma after liver transplantation. Transpl Infect Dis 2012;14:422–6. 22650490 10.1111/j.1399-3062.2012.00746.x

[3] Taylor AL Gibbs P Sudhindran S Monitoring systemic donor lymphocyte macrochimerism to aid the diagnosis of graft-versus-host disease after liver transplantation. Transplantation 2004;77:441–6. 14966423 10.1097/01.TP.0000103721.29729.FE

[4] Jamieson NV Joysey V Friend PJ Graft-versus-host disease in solid organ transplantation. Transpl Int 1991;4:67–71. 1910431 10.1007/BF00336399

[5] Norris S Collins C Doherty DG Resident human hepatic lymphocytes are phenotypically different from circulating lymphocytes. J Hepatol 1998;28:84–90. 9537869 10.1016/s0168-8278(98)80206-7

[6] Gulbahce HE Brown CA Wick M Graft-vs-host disease after solid organ transplant. Am J Clin Pathol 2003;119:568–73. 12710129 10.1309/395B-X683-QFN6-CJBC

[7] Luckraz H Zagolin M McNeil K Graft-versus-host disease in lung transplantation: 4 case reports and literature review. J Heart Lung Transplant 2003;22:691–7. 12821167 10.1016/s1053-2498(02)00811-2

[8] Zhang Y Ruiz P Solid organ transplant-associated acute graft-versus-host disease. Arch Pathol Lab Med 2010;134:1220–4. 20670147 10.5858/2008-0679-RS.1

[9] Kato T Yazawa K Madono K Acute graft-versus-host-disease in kidney transplantation: case report and review of literature. Transplant Proc 2009;41:3949–52. 19917421 10.1016/j.transproceed.2009.05.030

[10] Kim JM Kim SJ Joh JW Graft-versus-host disease after kidney transplantation. J Korean Surg Soc 2011;80 (suppl 1):S36–9. 22066080 10.4174/jkss.2011.80.Suppl1.S36 PMC3205370

[11] Ohtsuka Y Sakemi T Ichigi Y A case of chronic graft-versus-host disease following living-related donor kidney transplantation. Nephron 1998;78:215–7. 9496741 10.1159/000044914

[12] Smith DM Agura ED Levy MF Graft vs host disease following kidney transplantation using an ’0 HLA antigen mismatched’ donor. Nephrol Dial Transplant 2006;21:2656–9. 16627604 10.1093/ndt/gfl174

[13] Zacharias N Gallichio MH Conti DJ Graft-versus-host disease after living-unrelated kidney transplantation. Case Rep Transplant 2014;2014:971426. 24812587 10.1155/2014/971426 PMC4000646

[14] Taylor AL Gibbs P Bradley JA Acute graft versus host disease following liver transplantation: the enemy within. Am J Transplant 2004;4:466–74. 15023138 10.1111/j.1600-6143.2004.00406.x

[15] Matsukuma KE Wei D Sun K Diagnosis and differential diagnosis of hepatic graft versus host disease (GVHD). J Gastrointest Oncol 2016;7 (suppl 1):S21–31. 27034810 10.3978/j.issn.2078-6891.2015.036 PMC4783620

[16] Shulman HM Kleiner D Lee SJ Histopathologic diagnosis of chronic graft-versus-host disease: National Institutes of Health Consensus Development Project on Criteria for Clinical Trials in Chronic Graft-versus-Host Disease: II. Pathology Working Group Report. Biol Blood Marrow Transplant 2006;12:31–47. 10.1016/j.bbmt.2005.10.023 16399567

